# Late blight resistance of Julius Kühn Institute pre-breeding potato clones: a genome-wide association study

**DOI:** 10.1186/s12870-026-09266-3

**Published:** 2026-06-17

**Authors:** Johanna Blossei, Roman Gäbelein, Thilo Hammann, Julian Kirchgesser, Ralf Uptmoor

**Affiliations:** 1 Julius Kühn-Institute (JKI) - Federal Research Centre for Cultivated Plants, Institute for Breeding Research On Agricultural Crops, Sanitz, Germany; 2 Chair of Agronomy, Faculty of Agriculture and Environmental Science, University of Rostock, Rostock, Germany

**Keywords:** Late blight, *Phytophthora infestans*, *Solanum tuberosum*, Genome-wide association study

## Abstract

**Background:**

Late blight caused by *Phytophthora infestans* remains one of the most devastating diseases in potato cultivation, and durable resistance requires the combination of multiple resistance loci. The Julius Kühn Institute (JKI) has maintained a late blight resistance breeding program for more than 60 years and has developed extensive pre-breeding material with largely unknown resistance genetics. To characterize the genetic basis of this resistance, field trials were conducted over three years with 200 genotypes, including pre-breeding clones and cultivars. Genotypes were inoculated with *P. infestans* and assessed for foliage blight by relative area under the disease progress curve (rAUDPC) and maturity-corrected ΔrAUDPC. Detached leaf assays and tuber slice tests were evaluated as additional resistance-related traits.

**Results:**

Genome-wide association studies (GWAS) identified significant marker-trait associations for foliage and tuber blight resistance exclusively on chromosomes 9 and 11. The chromosome 9 locus accounted for up to 28% and the chromosome 11 locus for up to 16% of the marginal variance in phenotypic resistance, depending on the trait analyzed. Of the 200 genotypes, 121 carried the alternative allele on either chromosome and showed markedly reduced disease symptoms compared with non-carriers. Only one genotype carried resistance alleles at both loci, suggesting near-independent segregation and indicating good prospects for marker-assisted pyramiding.

On chromosome 11, 52 significant markers spanned an approximately 5.2 Mbp region. The significant markers themselves showed strong linkage disequilibrium (LD), consistent with a shared introgressed LD block harboring five NBS-LRR candidate genes. Two candidates are of particular interest, one located 13.8 kb from the most significant marker and another containing a significant marker within the gene.

**Conclusions:**

The major GWAS signal for late blight resistance in European potato breeding material on chromosome 11, with two structurally divergent NBS-LRR genes emerging as strong positional candidates, further supports this region as a resistance hotspot. Together with the second marker-trait association on chromosome 9, these results provide a useful genetic framework for the targeted pyramiding of resistance loci in potato breeding and support the development of cultivars with more durable late blight resistance.

**Supplementary Information:**

The online version contains supplementary material available at 10.1186/s12870-026-09266-3.

## Background

Potato cultivation is characterized by high yield losses from late blight, caused by the oomycete *P. infestans*, which remains the most devastating pathogen in this crop. Yield losses are particularly high in organic farming, where only copper formulations are permitted, while the use of synthetic chemical pesticides is prohibited. Infestation with late blight is manifested on the plant by brown spots on stems and leaves and a white mycelial layer on the underside of the leaves. Tubers show dark spots and become soft and mushy due to secondary infestation with bacteria [1]. In addition to agronomic measures, the cultivation of resistant cultivars is one of the most promising ways to prevent late blight. Resistance to *P. infestans* can be caused by individual resistance genes (*R* genes, qualitative) or quantitative trait loci (QTL, quantitative). In the mid-twentieth century, much work was invested in the utilization of resistance from *Solanum demissum*, in which 11 *R* genes were identified [2]. Unfortunately, it was shown that qualitative resistances like these 11 *R* genes can be rapidly overcome by pathogen adaptation [3–5].

In total, more than 70 *R* genes of 32 *Solanum* species have been identified and mapped to date [6]. Most of them originate from Mexican, Bolivian, or Peruvian wild species. Besides *S. demissum*, *S. bulbocastanum*, *S. berthaultii*, *S. stoloniferum*, *S. edinense*, *S. venturii*, *S. hjertingii*, and *S. chacoense* are also important sources for *R* genes. *R* genes predominantly encode nucleotide-binding site and leucine-rich repeat (NB-LRR) proteins and are clustered on chromosomes 1, 4, 5, 6, 7, 8, 9, 10, and 11. However, transferring resistance genes from wild relatives into cultivated potato is, e.g., hampered by differences in ploidy levels between most wild species and the tetraploid cultivated potato, and only a small number have been successfully introgressed into breeding material or released cultivars to date [6, 7].

Stacking of different *R* genes (*R*-gene pyramiding) is a promising way to obtain durable resistance in crops. As an alternative to time-consuming conventional breeding approaches, it is possible to transfer several *R* genes from crossable wild species to commercial cultivars by means of gene transfer (cisgenesis). This has already been proven successful several times [8, 9]. The disadvantage is that such plants are considered GMOs based on a 2018 decision by the Court of Justice of the European Union (CJEU, case C-528/16), and, thus, fall under EU genetic engineering legislation, making approval for cultivation very cumbersome for farmers in member countries. Hence, breeding cultivars with multiple *R* genes and/or QTL is likely to be an important route towards more durable resistance in new cultivars for the European market.

At JKI, resistant pre-breeding material has been developed for more than 60 years. Initially, crosses were made with resistant accessions of wild species. These were backcrossed up to seven times with cultivars to develop pre-breeding clones with improved agronomic performance. The gene pool at JKI was previously investigated for the presence of various *R* genes from wild species [10]. Some highly resistant breeding clones were identified, which carry none of the genes evaluated in the study above. Accordingly, it was hypothesized that QTL effects or other *R* genes likely also have a strong influence on the resistance in this material.

The aim of this study was to identify genetic loci associated with late blight resistance in potato pre-breeding material from JKI and the Bavarian State Research Centre for Agriculture (LfL), Freising, Germany by GWAS. In addition, a set of cultivars was included as reference material. By combining field trials, detached leaf assays and tuber slice tests with genotyping, we aimed to (i) characterize the resistance level of the material, (ii) detect marker-trait associations for foliage and tuber blight, and (iii) provide an additional basis for marker-assisted selection of clones with durable resistance.

## Material and methods

### Plant material and phenotypic data

The trials were carried out at the JKI's experimental field in Groß Lüsewitz, north-east Germany. For this purpose, 200 entries, including 161 pre-breeding clones and 39 cultivars (Additional file 1), were grown over three years in a randomized block design with two replications and ten plants per plot in each of the three years from 2019 to 2021. Means across years were used for GWAS.

The plant material comprised registered cultivars and pre-breeding clones from JKI, Groß Lüsewitz, Germany, and LfL, Freising, Germany. The *S. tuberosum* material used in this study was not collected from the wild but was either developed at the JKI or LfL or obtained as part of the project EffiKar, which was funded by the Federal Ministry of Food and Agriculture (BMEL), under the conditions of the Standard Material Transfer Agreement (SMTA) of the International Treaty on Plant Genetic Resources for Food and Agriculture. Several entries were donated directly by breeders under the same SMTA conditions. As the study used cultivated breeding material only, no permissions for wild collection were required, and voucher specimens were not applicable. All legal regulations concerning plant genetic resources were fulfilled. All methods were carried out in accordance with national and international guidelines for plant experiments. A complete list of all entries with full accession names is provided in Additional file 1.

Based on the taxonomic classification of Hawkes et al. [11], all wild species used for interspecific crosses belonged to the genus *Solanum*, section *Petota*, subsection *Potatoe*, and subseries *Stellata*, *Rotata*, and *Tuberosa*. The 161 higher generation backcross progeny of this study were derived by interspecific crosses between potato cultivars and different sexually compatible wild species as *S. demissum*, *S. phureja*, *S. andigena*, *S. stoloniferum*, *S. circaeifolium*, *S. spegazzinii*, *S. vernei*, and *S. okadae*. The interspecific hybrids were backcrossed four to six times with cultivated potato cultivars and selected in each backcross cycle for late blight resistance and agronomic traits. Each backcross cycle took seven years of intensive progeny testing. The wild species were received from the present-day named Groß Lüsewitz Potato Collections (GLKS) of the Leibniz Institute of Plant Genetics and Crop Plant Research (IPK), Gatersleben, Germany and its predecessor organizations.

At the end of flowering of the cultivar 'Adretta', one plant per experimental plot was inoculated with 5 ml of a *P. infestans* suspension of common field isolates [12], a composition which was supplemented each year with newly collected pathogen material (1.2 × 10^4^ sporangia ml^−1^) and tested on the differential set of Black [13].

The isolates were characterized via Flinders Technology Associates (FTA) cards by the James Hutton Institute, Dundee, UK. Between 2019 and 2022 the main late-blight strain was EU-41-A2. In 2020, a very small amount of other, not identified strains was found as well. However, EU-41-A2 is to date the main strain in northern Europe. Before artificial late-blight infection in field experiments, inoculum was collected from fields, which received no fungicide treatments and maintained at the JKI laboratory on potato tuber slices from a cultivar that has no resistance genes. Refreshing the inoculum every season with newly collected field material was done to ensure its high aggressiveness. The inoculum consistently showed virulence against the resistance genes *R1* to *R11* except the *R9/R9a* resistance specificities.

The infestation was assessed as the percentage of the infested foliage area of each plot, excluding the inoculated plant. This was repeated every three to four days until an infestation of 100% was reached, or until senescence. Based on these assessments, the relative area under the disease progress curves (rAUDPC) were calculated [14, 15]. Since late blight resistance is often associated with late maturity, rAUDPC values were additionally corrected for maturity as described in [16] and presented as ΔrAUDPC values. The ∆rAUDPC value corresponds to the residual of a linear regression of rAUDPC on the maturity score and was calculated as ∆rAUDPC = rAUDPC—rAUDPC_pred_, where rAUDPC_pred_ is the value predicted by the regression model. In addition to field testing, detached leaf assays were carried out and tuber slice tests were used in all trial years to investigate tuber blight resistance.

For the detached leaf assay, five uniform leaves of different plants per plot were taken shortly before inoculation in the field trial and 20 μl drops of the *P. infestans* suspension were applied onto the underside of each leaf in the laboratory. After 24 h, the leaves were turned and incubated for another five days at 16 °C, 95% relative humidity (RH), and 150 Lux. Scoring was based on the necrotic leaf area and mycelium formation on the underside of the leaf with scores from 1 (no infestation) to 9 (full infestation). The tuber slice test was performed on four tubers per plot. Slices from each tuber were inoculated with 20 μl of low-concentration (1,900 sporangia ml^−1^) and high-concentration (15,000 sporangia ml^−1^) *P. infestans* suspensions. After 24 h, the slices were rotated and subsequently incubated for another five days at 16 °C, 95% RH. Scoring was based on mycelium formation and browning of tuber flesh with scores from 1 (no infestation) to 9 (full infestation) for both concentrations. The mean value was used for evaluation.

The three assay types were used to capture complementary aspects of late blight resistance. Field-based rAUDPC and ΔrAUDPC integrate overall severity and disease progression rate under field conditions, with ΔrAUDPC correcting for maturity effects to separate resistance from escape due to early maturity. The detached leaf assay provides assessment of foliar resistance under controlled conditions, while the tuber slice test specifically targets tuber resistance, which may be governed by partially distinct mechanisms.

Pearson's correlation coefficients between year-specific trait values were calculated across all entries and heritabilities of the phenotypic traits were calculated with PLABSTAT version A [17] as described in [18].

### DNA extraction and genotyping

DNA was extracted from young leaves using the DNeasy Plant Pro Kit from Qiagen and sent to LGC Genomics GmbH. LGC performed genotyping by sequencing (GBS) with 2 × 150 bp (NextSeq 500/550 v2) and approximately 1.5 million reads per sample with the enzyme combination PstI-ApeKI. Reads were aligned to the potato reference genome GCA_000226075.1, strain DM1-3 516 R44 from the Potato Genome Sequencing Consortium (SolTub_3.0) [19].

A genomic relationship (kinship) matrix was calculated using the VanRaden method [20] as implemented in the R package AGHmatrix version 2.0.4 [21], based on 19,842 SNPs with ploidy set to 4. The matrix was used for cluster analysis performed using the neighbor-joining algorithm [22] as implemented in the R package phangorn [23]. Population structure was assessed using snmf in the R package LEA. Tetraploid marker dosages were simplified to diploid approximation to allow snmf analysis (0 = 0; 1,2 = 1; 3,4 = 2). The number of K subpopulations was evaluated using cross-entropy. Because no clear optimum was observed, K = 5 was selected as a pragmatic representation of the data.

### Genome-wide association study

GWAS were carried out with the R package GWASpoly [24]. For marker quality filtering, only markers with a minimum read depth of DP ≥ 20 and a call rate of at least 95% were retained. To preserve rare resistance alleles potentially introduced by wild species introgression, a minimum minor allele count of five individuals was applied instead of a conventional MAF threshold. The 310 SNPs with more than 4 alleles were removed. The number of markers in the final matrix for GWAS was 19,825. The kinship matrix was calculated using the leave-one-chromosome-out (LOCO) approach implemented in GWASpoly, to prevent the target chromosome from contributing to its own kinship correction. The first three principal components (PCs) derived from the marker data were included as covariates to account for population structure. The 1-dom-alt model was applied to all traits. This model assumes full dominance of the alternative allele, such that a single copy of the alternative allele is sufficient to confer resistance. Preliminary analyses confirmed that increasing allele dosage did not significantly affect resistance level, supporting the dominance assumption. Accordingly, a dosage model did not provide additional explanatory power compared to the 1-dom-alt model. The significance threshold was set at -log_10_(p) = 5.3, corresponding to the M.eff method [25] at α = 0.05.

In addition, log-transformed trait values (log1p) were evaluated in exploratory GWAS analyses to account for non-normal phenotypic distributions. These analyses resulted in largely consistent association patterns, with only minor differences in the number of detected loci. As no substantial improvement in model performance or biological interpretability was observed, results based on the original trait values are reported.

The explained percentage of phenotypic variance (R^2^) was calculated by regressing SNP marker dosage (0 = no alternative allele, 1 = one or more alternative alleles) on the resistance test results. These values represent marginal marker effects and do not account for population structure or relatedness and therefore may overestimate the proportion of variance explained.

Linkage disequilibrium (LD) was estimated as composite r^2^ as described in [26] with the R package ldsep [27], accounting for allele dosage in tetraploid genotypes. For the genome-wide LD decay analysis, 300 markers were randomly sampled per chromosome and all pairwise r^2^ values within each chromosome were computed. A LOESS curve (span = 0.1) was fitted across all chromosomes combined to visualize the decay pattern. For the Chr11 resistance region, LD was computed among all 353 markers within a ∼5.3 Mbp region (158,174—5,475,012 bp) encompassing the significant GWAS signals and providing a local estimate of LD structure around the candidate loci.

Differences in mean trait values among groups were assessed using linear models (lm) with group as a fixed factor for each of the four traits. Pre-breeding clones and cultivars were classified into groups based on the presence or absence of alternative alleles at the most significant independent GWAS markers. Estimated marginal means (EMMs) and pairwise contrasts were computed using the emmeans package with Tukey correction for multiple comparisons.

To test whether the significant markers on chromosome 11 represented a single locus or multiple independent loci, a conditional GWAS was performed using two approaches: First, the marker with the highest -log_10_(p) score on chromosome 11 was included as a fixed covariate in the GWASpoly model. Second, the effect of the same marker was removed from each phenotypic trait by linear regression of the trait on the allele dosage at this marker. The residuals of the regression were then used as the response variable in a second GWASpoly run with identical model specifications.

### Candidate gene analysis

Candidate genes within the Chr11 resistance region were identified using the *Solanum tuberosum* reference sequence v3.0 (SolTub_3.0, GCA_000226075.1) via Ensembl Plants (plants.ensembl.org). All annotated genes within the region defined by the significant markers were inspected for functional annotation, and NBS-LRR genes were identified as candidate resistance genes. To obtain corresponding gene identifiers in the current reference genome version, the coding sequences of all five NBS-LRR candidate genes were aligned against the *S. tuberosum* v6.1 gene models [28] (spuddb.uga.edu) using BLASTN version 2.2.26. For protein characterization, the predicted protein sequences of the two top candidates were retrieved from NCBI RefSeq (XP_006353282.1 and XP_015166427.1) and their domain architecture was inspected using the NCBI Conserved Domain Database (CDD). To assess sequence similarity to known late blight resistance proteins, pairwise BLASTP alignments were performed between each candidate protein and four well-characterized Rpi proteins originally derived from *S. demissum*: R1 (Q8W1E0), R2 (ACU65456), R3a (AAW48299), and R3b (AEC47890).

## Results

**Fig. 1:**
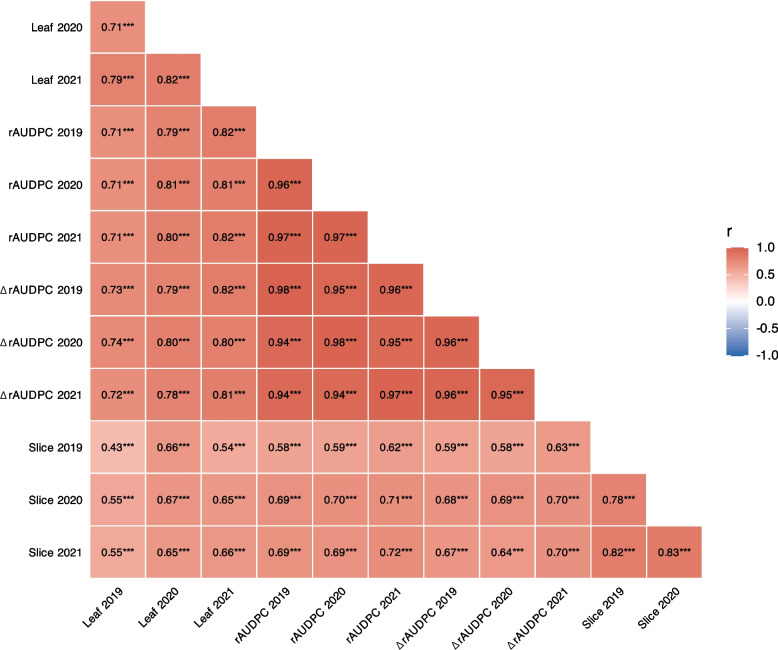
Pearson's correlation coefficients between relative area under disease progress curve (rAUDPC), the maturity-corrected ∆rAUDPC, detached leaf assay scores (Leaf), and the tuber slice tests (Slice) of three years field and laboratory trials

Phenotypic data showed that the cultivars with mean values of 5.6 for the detached leaf assay, 0.717 for the rAUDPC values, 0.482 for the ∆rAUDPC values, and 5.0 for the tuber slice test were much more susceptible to late blight than the pre-breeding clones with mean values of 2.0 for the detached leaf assay, 0.107 for the rAUDPC values, −0.079 for the ∆rAUDPC values and 2.7 for the tuber slice test (Additional file 1). Inter-year correlations were high for all traits (*r* = 0.71–0.97), indicating limited entry-by-year interactions (Fig. 1). Overall, there was a very high correlation between the results of the field test and the detached leaf assay. The correlation between the tuber slice test and tests for foliage blight was lower, but still significant.

Of the 161 pre-breeding clones, 134 of showed high late blight resistance with ∆rAUDPC values < 0. The remaining 27 clones were susceptible with ∆rAUDPC values ≥ 0. Of the 39 cultivars, only 'Sarpo Mira', 'Bionica', and 'Toluca' were considered to be resistant according to their ∆rAUDPC values < 0. However, entries with ∆rAUDPC values < 0 are not necessarily fully resistant. Among the cultivars, 'Sarpo Mira' had the lowest susceptibility; 'Bionica' and 'Toluca' could also be described as moderately susceptible.

The broad-sense heritability for foliage late blight resistance was 0.76 for the rAUDPC values, 0.73 for the ∆rAUDPC values and 0.68 for the detached leaf assay scores. The heritability for tuber blight resistance was 0.67.

**Fig. 2:**
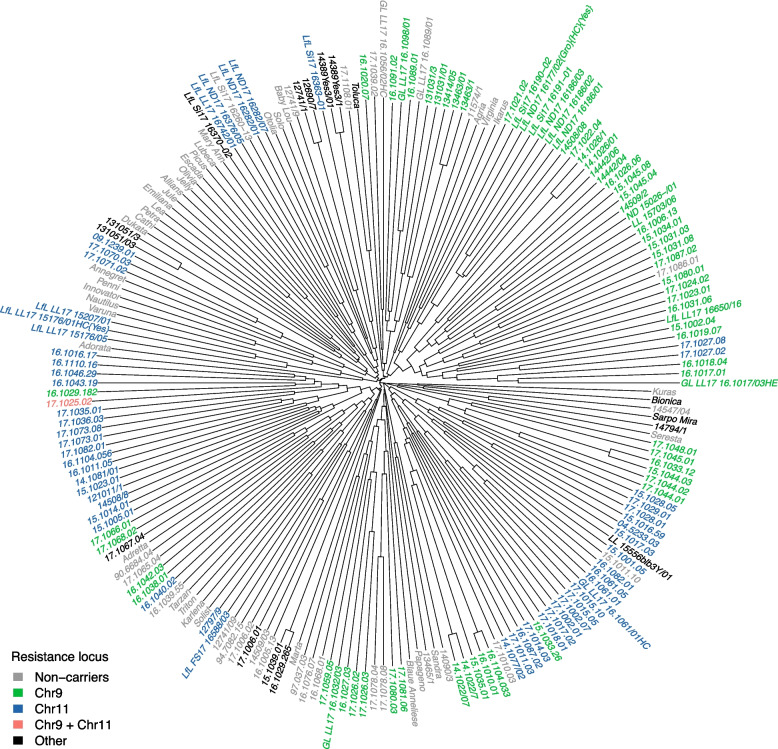
Neighbor-joining tree of 200 pre-breeding clones and cultivars based on a genomic relationship (kinship) matrix. Branch tip colors indicate resistance locus classification based on GWAS results

The neighbor-joining tree based on the genomic relationship matrix revealed no distinct cluster structure within the studied panel. Pre-breeding clones and cultivars were distributed across the tree without forming clearly separated groups, reflecting the diverse composition of the breeding material (Fig. 2). LEA snmf confirmed the absence of distinct population clusters. Cross-entropy across K = 2—12 showed no clear optimal number of K subpopulations. At both K = 5 and K = 8, approximately 75% of the entries were classified as admixed (max. ancestry proportion < 0.7). At K = 5, resistant entries (∆rAUDPC < 0) were distributed across all groups, with 95% classified as resistant in groups 2 and 5 and between 31 and 54% in groups 1, 3, and 4 (Additional files 1 and 2).

**Fig. 3:**
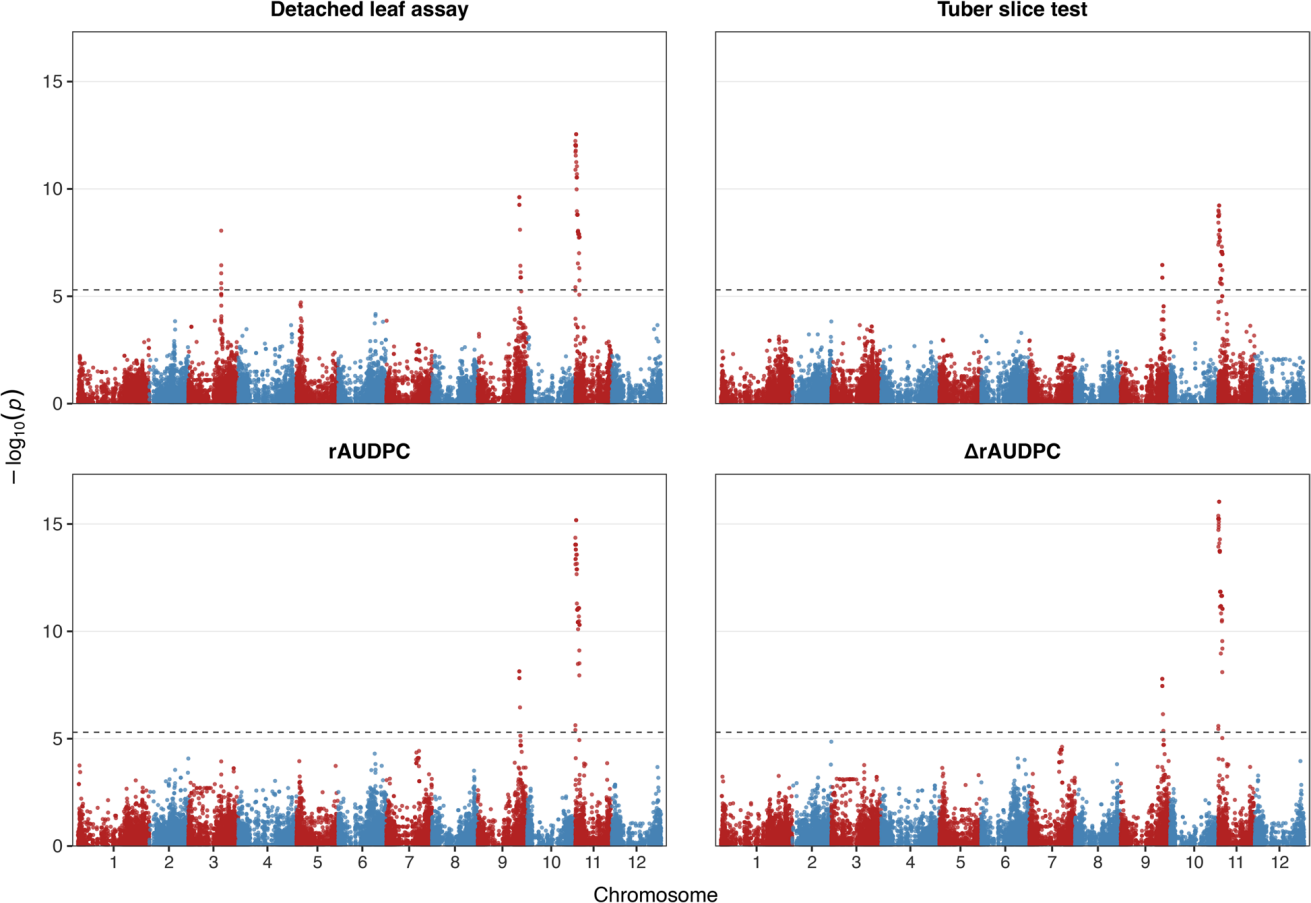
Manhattan plots for the detached leaf assay, tuber slice test, rAUDPC, and maturity-corrected relative area under disease progress curve (∆rAUDPC)

GWAS revealed five significant marker-trait associations on chromosome 3 only for one trait (detached leaf assay). However, the alternative allele at these markers was present in the majority of entries (142—178), indicating near fixation within the population. Since these associations provided little discriminatory power for resistance because the alternative allele was present in most entries, these markers were not considered further. An additional ten significant marker-trait associations for the leaf assay were located on chromosome 9 (Chr9) and 50 markers significantly associated with the detached leaf assay on chromosome 11 (Chr11) (Fig. 3, Additional file 3). For the tuber slice test, there were four significant associations on Chr9 and 47 on Chr11. Broadly consistent with these findings, we identified five significant marker-trait associations for rAUDPC and six for ∆rAUDPC on Chr9 (Additional file 3). The number of significant associations on Chr11 for both traits was 52. All traits had the highest -log_10_(p) value on markers 11_1123733 and 11_1214801. The overall maximum -log_10_(p) score was 16.04 for ∆rAUDPC.

**Fig. 4:**
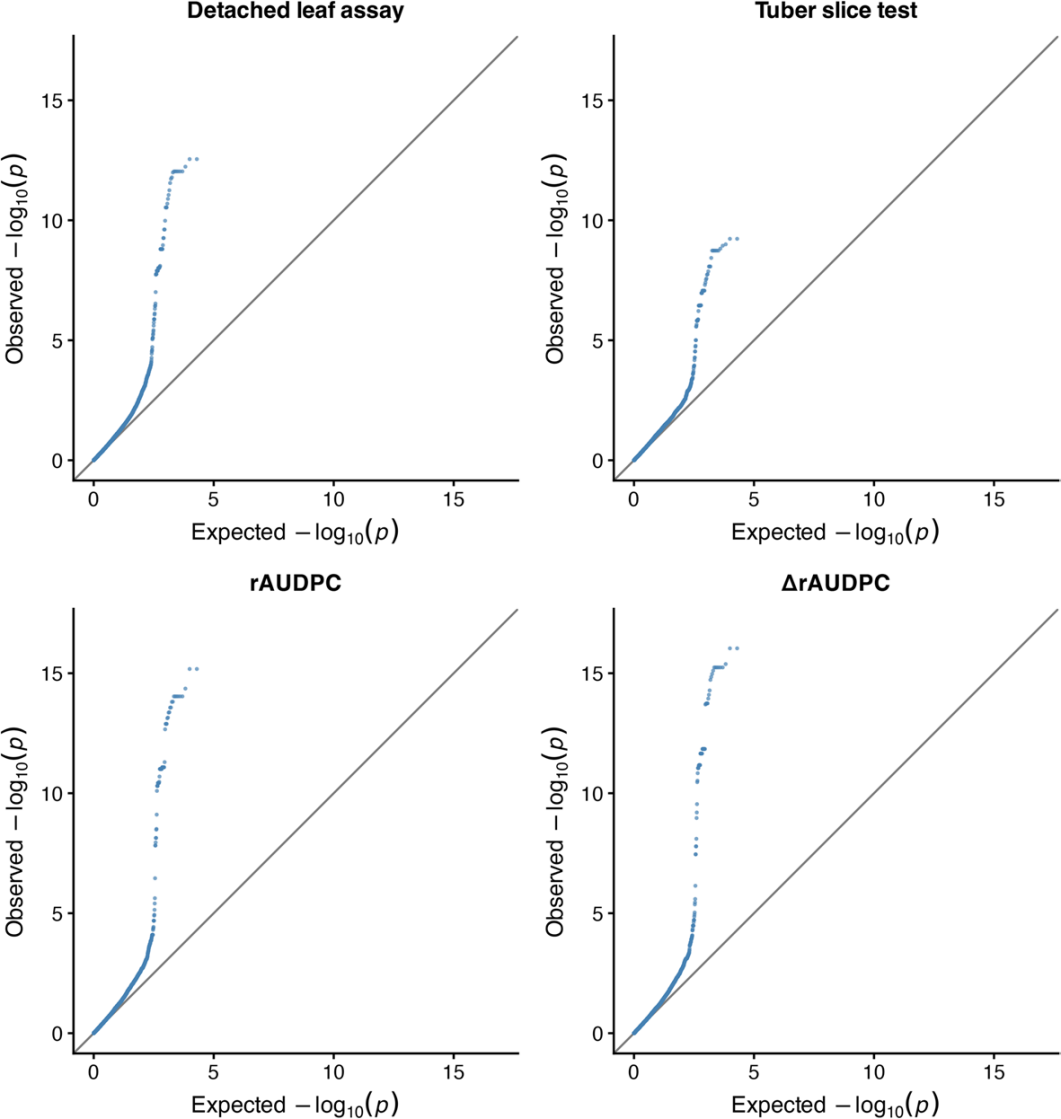
QQ plots for the detached leaf assay, tuber slice test, rAUDPC, and maturity-corrected relative area under disease progress curve (∆rAUDPC)

QQ plots for all four traits showed that the observed -log₁₀(p) values followed the expected distribution closely along the diagonal up to -log₁₀(p) = 3, suggesting that the applied correction adequately controlled the broad population structure. A very clear deviation from the null hypothesis was observed for all traits in the upper tail, which is consistent with the presence of true marker-trait associations at the identified resistance regions on Chr9 and Chr11 (Fig. 4).

Results of the most significant markers on Chr9 and Chr11 are summarized in Table 1. Complete results are provided in Additional file 3. A total of 78 entries (all 39 cultivars and 39 pre-breeding clones) did not carry an alternative allele at either of the two top markers on Chr9 and Chr11. Of these 39 clones, 26 showed ∆rAUDPC values ≥ 0 (Additional file 1). On the marker locus at 51,556,194 bp on Chr9, 67 pre-breeding clones carried at least one alternative allele putatively associated with late blight resistance. On the locus at 1,214,801 bp on Chr11, 54 clones carried at least one alternative allele. Only one clone carried alternative alleles on both chromosomes. The near-absence of double carriers may reflect the breeding history of the material but it should be noted that this observation is based on the two most significant markers per chromosome. This does not exclude the possibility of additional co-occurrences across the full set of significant markers.

**Table 1:** Most significant GWAS markers per trait and chromosome identified with the 1-dom-alt model. Effect = allele substitution effect. R^2^ = phenotypic variance explained

Trait	Chromosome	Position (bp)	Ref	Alt	-log_10_(p)	Effect	R^2^ (%)
Detached leaf assay	9	51,556,194	A	G	9.61	−2.72	27.9
	11	1,214,801	T	C	12.55	−2.58	11.3
Tuber slice test	9	51,556,194	A	G	6.46	−1.50	28.0
	11	1,214,801	T	C	9.23	−1.47	5.30
rAUDPC	9	51,556,194	A	G	8.14	−39.04	20.7
	11	1,214,801	T	C	15.18	−43.73	13.9
ΔrAUDPC	9	51,556,194	A	G	7.79	−0.36	19.7
	11	1,214,801	T	C	16.04	−0.42	15.8

Pre-breeding clones carrying resistance loci on Chr9 or Chr11 were distributed across the entire neighbor-joining tree, largely without forming distinct clusters, indicating that the resistance loci are not strongly confounded with the genealogical structure of the panel (Fig. 2).

Although marker effects of 11_1214801 were higher for rAUDPC and ΔrAUDPC compared to the effects of 9_51556194, the phenotypic variance explained from the marker-trait association on Chr9 was higher for all traits, which reflects the larger number of pre-breeding clones carrying the resistance allele on Chr9 (Table 1). The reported R^2^ values should be interpreted as indicative effect sizes rather than precise estimates of genetic variance explained.

**Fig. 5:**
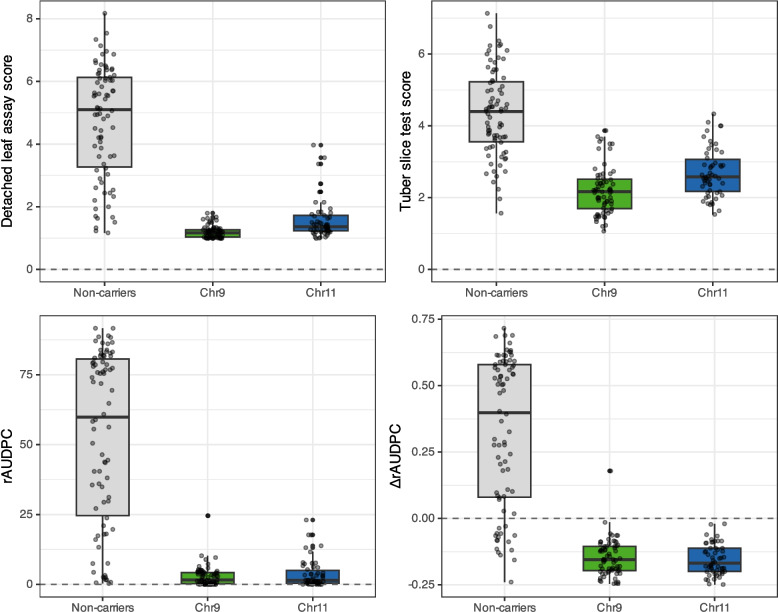
Boxplots for the scores of the detached leaf assay, the tuber slice test, rAUDPC, and ∆rAUDPC divided into the groups carrying at least one alternative allele on the position with the highest -log_10_(p) values on chromosomes Chr11 and Chr9 or carrying neither alternative alleles on these two positions (Non-carriers)

Based on the GWAS results, entries were assigned to three groups: (i) Chr11: carriers of at least one alternative allele at Chr11 marker 11_1214801 but not at Chr9, (ii) Chr9: carriers of at least one alternative allele at marker 9_51556194 but not at Chr11, (iii) Non-carriers: entries carrying no alternative allele at either locus. One genotype carrying alternative alleles at both loci was excluded from group-based analyses to avoid confounding. Overall, entries carrying at least one alternative allele were found to be clearly more resistant than non-carriers (Table 2, Fig. 5).

**Table 2:** Estimated marginal means (EMM), standard errors (SE) and confidence limits (CL) for the three groups Chr9, Chr11, and Non-carriers for the four traits from field and laboratory analysis

Trait	Group	EMM	SE	Lower CL	Upper CL	Tukey HSD
Detached leaf assay	Non-carriers	4.71	0.13	4.45	4.97	a
	Chr9	1.120	0.14	0.91	1.48	b
	Chr11	1.59	0.16	1.27	1.90	b
Tuber slice test	Non-carriers	4.36	0.10	4.15	4.56	a
	Chr9	2.20	0.11	1.99	2.43	b
	Chr11	2.70	0.13	2.45	2.90	c
rAUDPC	Non-carriers	52.64	2.24	48.23	57.05	a
	Chr9	2.91	2.41	−1.85	7.67	b
	Chr11	3.79	2.69	−1.51	9.10	b
ΔrAUDPC	Non-carriers	0.33	0.02	0.29	0.37	a
	Chr9	−0.15	0.02	−0.19	−0.10	b
	Chr11	−0.156	0.03	−0.20	−0.11	b

^*^Within traits, means followed by the same letter do not differ significantly (*p* ≥ 0.05)

**Fig. 6:**
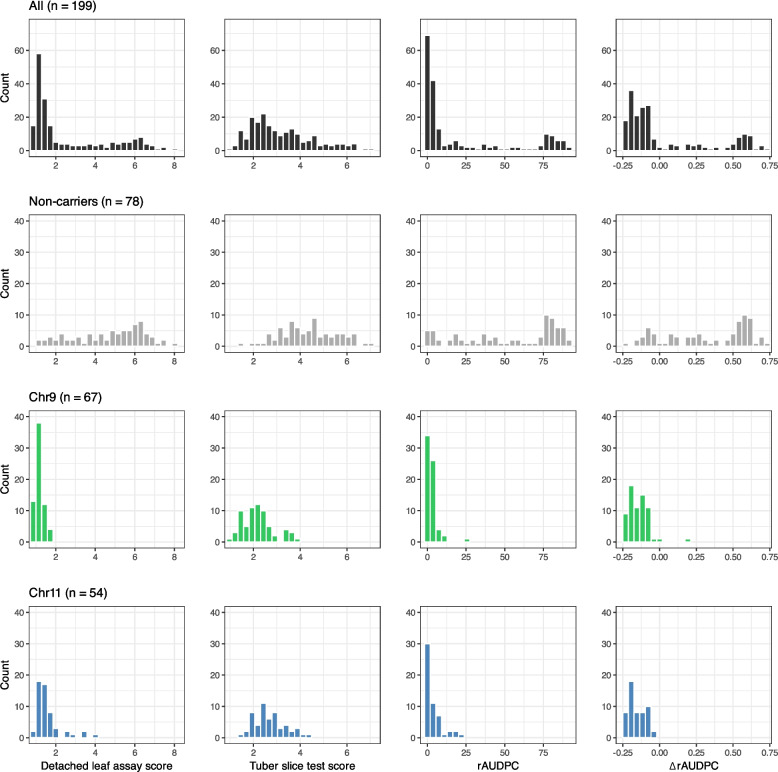
Frequency distributions of mean phenotypic values for detached leaf assay score, tuber slice test score, rAUDPC, and ΔrAUDPC across all entries and stratified by the groups Non-carriers, Chr9, and Chr11

Phenotypic distributions were non-normal due to the predominantly resistant composition of the pre-breeding material (Fig. 6). Residual diagnostics indicated heteroscedasticity among groups (Additional file 4), with the Non-carriers group showing greater residual variance than Chr9 and Chr11 carriers. However, the linear model was retained because group differences were pronounced, and the conclusions were supported by non-parametric analyses, as an additional Kruskal–Wallis test with Dunn's pairwise mean comparison and Bonferroni correction yielded results consistent with the parametric analysis and confirmed the robustness of the observed group differences. The larger residual variance observed in the Non-carriers group is consistent with the biological heterogeneity of this group, which comprises entries with diverse genetic backgrounds and potentially unknown resistance mechanisms.

**Fig. 7:**
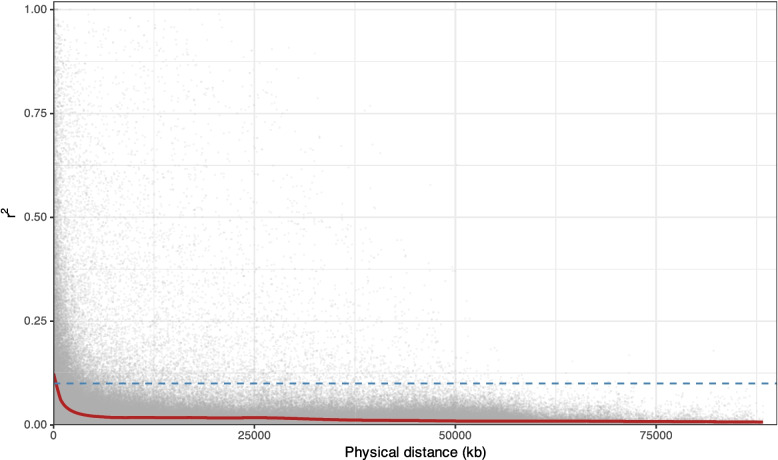
Genome-wide linkage disequilibrium (LD) decay across all 12 chromosomes. Each point represents an r^2^ value for a pairwise marker comparison. The red line shows the LOESS-smoothed decay curve fitted across all chromosomes combined. The dashed blue line indicates the r^2^ = 0.1 threshold

LD decay was computed across an extended ∼5.3 Mbp region encompassing all 52 significant markers on Chr11 (Additional file 5). After filtering, the resistance region carried 353 markers (6.6 markers/100 kb). Results revealed an overall low LD in the region with a rapid decay. The LD baseline was at r^2^ = 0.12, with the LOESS estimated r^2^ falling below 0.1 at approximately 53 kb and reaching its half-value (r^2^ = 0.058) at 661 kb. The majority of marker pairs showed low LD (median r^2^ = 0.011), with only a small percentage of marker pairs exceeding r^2^ = 0.2. Genome-wide LD decay across all 12 chromosomes was consistent with this pattern. The LOESS baseline ranged from r^2^ = 0.10 to 0.16 depending on the chromosome, with the half-value distance ranging from approximately 600 to 1,200 kb, and a genome-wide median r^2^ of 0.006 (Fig. 7).

**Fig. 8:**
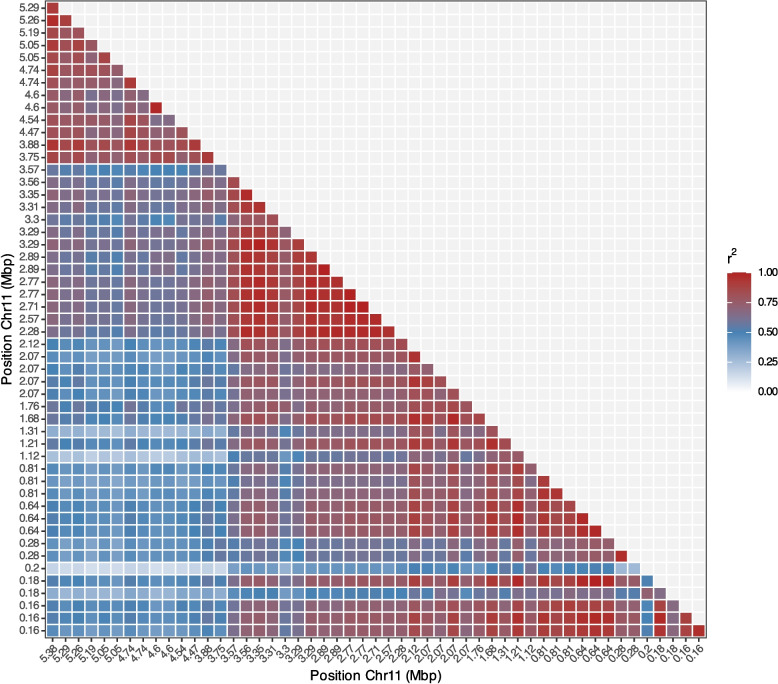
Pairwise linkage disequilibrium (r^2^) among the 52 significant Chr11 markers identified by GWAS. Marker positions (Mbp) are shown on both axes

In contrast to the generally low LD across the region, the significant markers themselves showed strong pairwise LD (Fig. 8). Most significant markers within the ∼5.2 Mbp region on Chr11 were strongly correlated with each other. Mean r^2^ across all pairwise marker combinations was 0.63, which is unusually high for this physical distance. This pattern is consistent with a shared LD block carried by a subset of genotypes. Of the 52 significant markers, 33 showed r^2^ ≥ 0.7 to the anchor marker at 1.21 Mbp. They were distributed uniformly across the region from 0.16 to 3.57 Mbp. LD declined gradually beyond 3.57 Mbp, indicating weaker linkage towards the distal part of the associated interval. The marker 11_200283 at 0.2 Mbp showed a distinct allele frequency pattern, with 74 carriers including 39 genotypes with dosage > 1, whereas most other Chr11 markers had 35—55 carriers and dosages ≤ 2.

Both conditional GWAS approaches yielded consistent results. After conditioning on the top Chr11 marker, the chromosome 11 association largely disappeared, supporting the interpretation that the significant markers reflect one major underlying signal. No evidence for an additional independent effect was detected in the distal region between 3.75 and 5.38 Mbp (Additional file 6). The Chr9 signal persisted and was slightly strengthened after conditioning.

The six significant markers for ΔrAUDPC on chromosome 9 cluster within a region of approximately 1.4 Mbp (51.56—52.96 Mbp). According to the *S. tuberosum* reference sequence v3.0 (plants.ensembl.org), no previously annotated late blight resistance genes co-localize directly with the significant marker interval on chromosome 9. Among the genes in the vicinity of the most significant markers, an ankyrin repeat-containing protein at 51.48 Mbp and an aldo–keto reductase at 51.54 Mbp are noteworthy. The latter is in close proximity to the top marker 9_51556194. While ankyrin repeat domains occur in some resistance-associated proteins and aldo–keto reductases have been implicated in stress responses, neither represents a classical *R*-gene family.

The LD profile of the most significant marker on Chr9, 9_51556194 shows a small peak at the GWAS signal, with several markers maintaining moderate r^2^ values extending to approximately 56 Mbp distally, beyond which LD declines, but not to background levels (Additional file 7). The v3.0 reference genome contains four genes annotated as late blight resistance proteins located at 46.45 Mbp, 54.64 Mbp, 59.41 Mbp, and 59.55 Mbp. The nearest markers to all four annotated genes showed no substantial LD with the top GWAS marker (r^2^ ≤ 0.06), and none can therefore be identified as a direct candidate gene.

Chromosome 11 harbors 54 annotated NBS-LRR loci, representing one of the highest NBS-LRR densities in the potato genome [29]. According to the *S. tuberosum* reference sequence v3.0, five NBS-LRR genes map within the Chr11 resistance region (Table 3). The distances between these genes and the nearest significant marker range from 0 to 102 kb. Two candidates are of particular interest: PGSC0003DMG400013308 is located at 1.20 Mbp, only 13.8 kb from the top Chr11 marker, and PGSC0003DMG400044423 at 2.71 Mbp contains the significant marker 11_2712959 within the candidate gene itself.

**Table 3:** Candidate genes in the Chr11 resistance locus of the *Solanum tuberosum* v3.0 reference genome. The v6.1 gene ID is shown in brackets

Gene ID v3.0 (v6.1)	Annotation	Position (Mbp)	Nearest marker	Distance (kb)
PGSC0003DMG400013308 (Soltu.DM.11G001680.1)	NBS-LRR	1.20	11_1214801	13.8
PGSC0003DMG401015682 (n.d.)	NBS-like 25	1.80	11_1755443	49.3
PGSC0003DMG400046545 (Soltu.DM.11G003310.1*)	NBS-LRR	2.54	11_2569425	26.7
PGSC0003DMG400044423 (Soltu.DM.11G003490.1)	NBS-LRR	2.71	11_2712959	0
PGSC0003DMG400027325 (Soltu.DM.11G004930.1)	BED-finger NBS-LRR	4.37	11_4473431	102.3

n.d. = not determinable, no unambiguous v6.1 ortholog identified

^*^partial match only, v3.0 sequence maps to multiple v6.1 loci

PGSC0003DMG400044423 is located on the minus strand of chromosome 11 at 2,710,023—2,713,943 bp and encodes an NBS-LRR resistance protein. The SNP represents a synonymous substitution at the coding sequence position 2337, codon 779 (AGC → AGT), with no predicted change at the amino acid level (Ser → Ser). Synonymous mutations are generally considered neutral, but they may still affect gene expression. Accordingly, the primary significance of this SNP lies in its utility as a closely linked marker for the underlying resistance locus rather than as a causative variant itself.

The two primary candidate genes were further characterized at the protein level. PGSC0003DMG400044423 is predicted to encode a full-length RPM1-like CC-NBS-LRR protein of 906 amino acids with an RX-type coiled-coil domain, an NB-ARC domain, and an LRR domain (RefSeq: XP_006353282.1). The gene appears to be intronless and is located on the reverse strand. PGSC0003DMG400013308 is predicted to encode an NB-LRR protein of 982 amino acids with NB-ARC and LRR domains. It is annotated as a putative inactive disease susceptibility protein LOV1 isoform X2 (RefSeq: XP_015166427.1). Pairwise BLASTP alignment of both proteins against the four well-characterized *S. demissum*-derived late blight resistance proteins R1, R2, R3a, R3b revealed sequence identities ranging from 24.4 to 27.7% with query coverages of 69 to 95%.

## Discussion

In the present study, pre-breeding potato clones from JKI and common cultivars were tested for their *P. infestans* resistance. Of the cultivars, only 'Sarpo Mira', 'Bionica', and 'Toluca' were classified as resistant based on their ∆rAUDPC values. The resistance genes *R3a*, *R3b*, R4, *Rpi-Smira1* and *Rpi-Smira2/R8* were found to be present in 'Sarpo Mira' [30–32]. 'Bionica' carries the resistance genes *R2*, *R3a*, *R3b*, and *Rpi-blb2*. In 'Toluca' the genes *R3a* and *Rpi-blb2* were identified [33, 34]. In addition to these known resistance genes, 'Sarpo Mira' carries at least one alternative allele at five significant markers distributed across the Chr11 resistance region at 0.16, 0.20, 0.81, 2.07, and 4.74 Mbp, which may reflect a fragmented wild species introgression. However, because alternative alleles were absent at the most strongly associated loci, this pattern could also represent random background variation. Neither 'Bionica' nor 'Toluca' carried alternative alleles at any of the significant GWAS markers, suggesting that their resistance is conferred by other loci not detected in this study.

Significant correlations were observed between all tests for foliage and tuber blight. For the correlation of the foliage blight tests with the tuber slice tests, this is in contrast to previous reports, as independent inheritance of the two traits is described in the literature [35–37]. Although correlations between foliage and tuber blight were lower than those between the different field and laboratory foliage blight tests in the present study, they do not support strictly independent inheritance within our population.

GWAS revealed significant marker-trait associations predominantly on chromosomes 9 and 11 under the assumption of a full dominance effect if at least one alternative allele was present. The number of alternative alleles did not significantly affect the resistance level, and a dosage model did not provide additional explanatory power compared to the 1-dom-alt model. The two resistance loci on chromosomes 9 and 11 segregated almost independently in the studied population, with only one genotype carrying alternative alleles at both top markers. This near-complete absence of co-occurrence is consistent with largely independent introgression events. Pyramiding both resistance loci into elite breeding clones would be a promising strategy to increase the durability of resistance against *P. infestans*.

Of the 200 genotypes, 16 showed low disease scores despite lacking alternative alleles at the top markers on Chr9 and Chr11, suggesting that additional resistance mechanisms not detected by this GWAS may segregate in the panel.

The well-known *R*-genes *R8* and *R9a* on chromosome 9 are on the same arm as the significant markers, but with around 8 Mbp [38–40] still too far away to be related to the marker-trait associations. However, a marker-trait association that is only 0.28 Mbp away from the one on chromosome 9 of the present study has been reported in [41].

To verify the genomic context of the Chr9 signal in the current reference genome, the syntenic region was identified in v6.1 using a conserved *Yellow-Stripe like* gene (PGSC0003DMG400011396/Soltu.DM.09G023330.1) as an anchor. The significant SNP 9_52489154 is located within the gene, which maps in v6.1 at 59.2 Mbp. Several annotated NBS-LRR and NB-ARC domain-containing genes in v6.1 formed a cluster located approximately 3.9—7.8 Mbp distal to the anchor (Soltu.DM.09G027060.1, Soltu.DM.09G029410.1, Soltu.DM.09G029670.1, Soltu.DM.09G029720.1, Soltu.DM.09G031120.1, and Soltu.DM.09G031250.1), suggesting that the Chr9 GWAS signal does not co-localize with known resistance gene clusters in either genome version. Because the v6.1 reference genome may not capture all donor-specific resistance haplotypes, this does not exclude the presence of an uncharacterized resistance gene or regulatory factor in the region. Fine-mapping and functional studies will be required to identify the causal gene underlying the resistance locus on chromosome 9.

The entire resistance region on Chr11 spans 5.2 Mbp. It was demonstrated that introgressed haploblocks in tetraploid potato may show substantially reduced LD decay compared to modern cultivars, with haploblock lengths several times larger in introgressed regions [42]. The extent and coherence of the Chr11 resistance region in the present study is consistent with the LD pattern expected for an introgressed segment. However, the reduced recombination within introgressed segments would impede fine-mapping of individual resistance genes and complicate the separation of favorable from unfavorable alleles in breeding for late blight resistance.

The overall low LD in the Chr11 hotspot region may reflect the genetic diversity of the studied population, in which the introgressed haplotype is present in only a subset of genotypes, diluting pairwise LD estimates across the full marker set. The coexistence of low overall LD in the region and strong LD among significant markers is consistent with a region where historical recombination has eroded linkage across most of the interval, while selection has preserved a conserved LD block in a subset of resistant genotypes.

Pairwise BLASTP alignments of both candidate proteins against well-characterized *S. demissum* derived resistance proteins revealed sequence identities of 24.4–27.7%. That is consistent with the background similarity observed among the reference Rpi proteins themselves, which share approximately 24 to 29% pairwise identity, with the exception of R3a and R3b, which constitute a closely related gene pair with 67% identity. The absence of elevated sequence similarity does not suggest a close relationship to the characterized Rpi proteins. Functional characterization, including expression analysis under *P. infestans* challenge and allelic diversity assessment across the study panel, will be required to determine which gene in the cluster underlies the observed association signal.

Several *R* genes and QTL against *P. infestans* from wild species have already been detected on chromosomes 9 and 11 in previous studies (Additional file 8, [29, 35–37, 41, 43–65]). The resistance locus on chromosome 9 identified in this study maps to the same chromosomal arm as the *R* genes *Rpi-moc1*, *Rpi-vnt1*, *R8/Rpi-Smira2*, *R9a,* and *Ph-3*, though these are located approximately 8 Mbp distal to the significant markers and are therefore unlikely to be directly related to the locus described here.

Previous studies have demonstrated that most of the *R* genes from *S. demissum* are located at the end of chromosome 11 [65], so that they are most likely not related to the marker-trait association detected in the present study. Besides *S. demissum*, also *S. phureja* was crossed with JKI material, so that the marker-trait association on chromosome 11 may originate from *S. phureja*. The *R* gene *Rpi-Smira1* from 'Sarpo Mira', which was also used for many crosses at JKI, is located on chromosome 11 as well. However, *Rpi-Smira1* is located close to the gene *R3a* from *S. demissum* [30], which is not located close to the significant markers of the present study. This is further supported by the conditional GWAS, which did not provide evidence for an additional independent effect in the distal region and is therefore consistent with the interpretation that the Chr11 association is largely driven by the proximal signal. In partial accordance, a QTL explaining 15.6% of phenotypic variance in a tetraploid potato panel was identified at 5.18 Mbp on chromosome 11 (reference genome v6.1) [43].

## Conclusion

This genome-wide association study of 200 potato genotypes identified two major resistance-associated regions on chromosomes 9 and 11. Markers at the Chr9 region accounted for up to 28% and those at the Chr11 region for up to 16% of the marginal phenotypic variance in late blight resistance. On chromosome 11, 52 significant markers spanned an approximately 5.2 Mbp region and showed strong LD among significant markers, consistent with a shared introgressed haplotype.

The two loci segregated almost independently in the studied population, with only one genotype carrying resistance alleles at both loci. This pattern suggests favorable conditions for pyramiding resistance-associated alleles through marker-assisted selection and represents a promising strategy for improving the durability of late blight resistance in potato breeding.

Five NBS-LRR candidate genes mapped within the Chr11 resistance region. Among these, PGSC0003DMG400013308, located 13.8 kb from the most significant marker and PGSC0003DMG400044423, containing a significant marker within the gene, represent particularly strong positional candidates for functional validation. No classical *R* gene is located in the Chr9 resistance region but the nearest known association from an independent study is only 0.28 Mbp away [38], supporting the biological relevance of this locus.

Future research should focus on sequencing and functional characterization of the NBS-LRR candidate genes, as well as gene expression analyses in *P. infestans*-inoculated tissues to elucidate the molecular basis of resistance at both loci. Overall, these results provide a useful genetic framework for marker-assisted pyramiding of resistance loci and support the development of potato cultivars with more durable late blight resistance.

## Supplementary Information

Additional file 1: Table S1: Phenotypic data and reference/alternative alleles at the most interesting SNP markers of the 161 pre-breeding clones and 39 cultivars used for the GWAS. Dominant ancestry groups, group assignment (max. ancestry proportion ≥ 0.7), and max. ancestry according to LEA snmf with K = 5.

Additional file 2: Figures S1, S2: Neighbor-joining tree colored by LEA snmf ancestry groups (K = 5) and the corresponding structure plot.

Additional file 3: Table S2: Significant marker-trait associations of the 1-dom-alt GWAS model.

Additional file 4: Figures S3—S6: Residual diagnostics for the detached leaf assay, tuber slice test, rAUDPC, and ∆rAUDPC fitted separately for each genotype group (Non-carriers, Chr9, Chr11). For each combination, histograms of residuals (top), residuals versus fitted values (middle), and quantile–quantile plots (bottom) are shown.

Additional file 5: Figure S7: Linkage disequilibrium (r^2^) decay across the Chr11 resistance region (0.16–5.48 Mb) among 353 markers. The LOESS curve (red line) illustrates the decay trend.

Additional file 6: Figures S8, S9: Manhattan plots after including 11_1214801 as a fixed covariate (first page) and after removing the marker effect from each phenotypic trait by linear regression (second page) for the traits detached leaf assay, tuber slice test, rAUDPC, and maturity-corrected relative area under disease progress curve (∆rAUDPC).

Additional file 7: Figures S10, S11: LD profiles of the most significant markers on chromosomes 9 and 11 (indicated by dashed orange lines) against all chromosome 9 and 11 markers, respectively. Each point represents the r^2^ value between the anchor marker and another chromosome 9 or 11 marker. The red line shows a LOESS-smoothed trend.

Additional file 8: Table S3: R-genes and QTL against late blight on chromosome 9 and 11 with the corresponding literature references.

## Data Availability

All data used here are included in the manuscript or in the Online Resources. The raw data can be provided on request from the corresponding author. Planting material and DNA samples of all entries of this study are deposited at JKI and available upon request for scientific purposes.
