# Supplementary material for: Late blight resistance of Julius Kühn Institute pre-breeding potato clones: a genome-wide association study
Source: BMC Plant Biol. 2026 Jun 17;26:1046. doi: 10.1186/s12870-026-09266-3 (PMC13273995; doi:10.1186/s12870-026-09266-3)
Supplement: Supplementary file 2 — Additional file 2: Figures S1, S2: Neighbor-joining tree colored by LEA snmf ancestry groups (K = 5) and the corresponding structure plot. [file 12870_2026_9266_MOESM2_ESM.pdf]

Neighbor joining tree coloured by LEA snmf ancestry groups (K = 5)

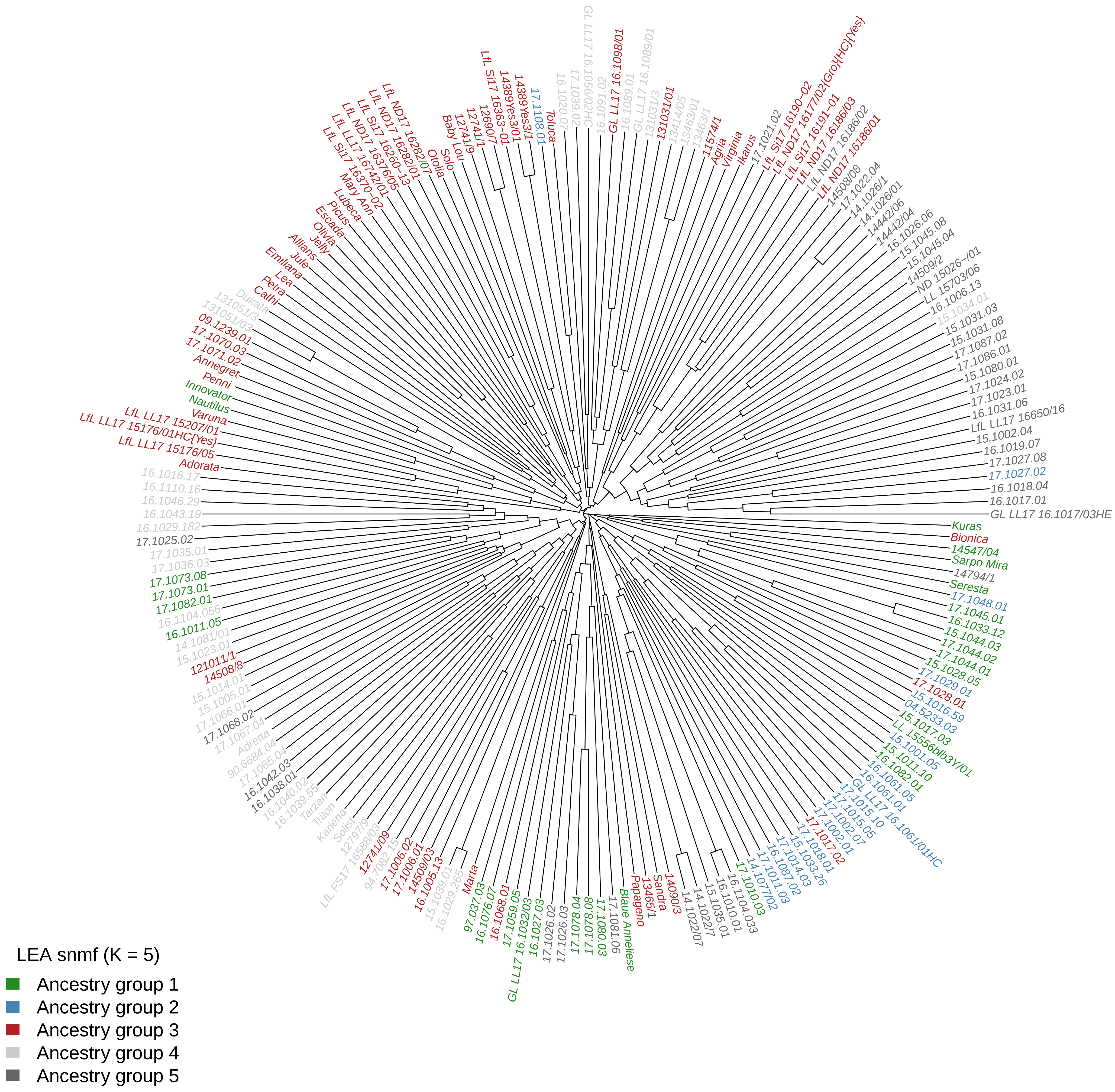

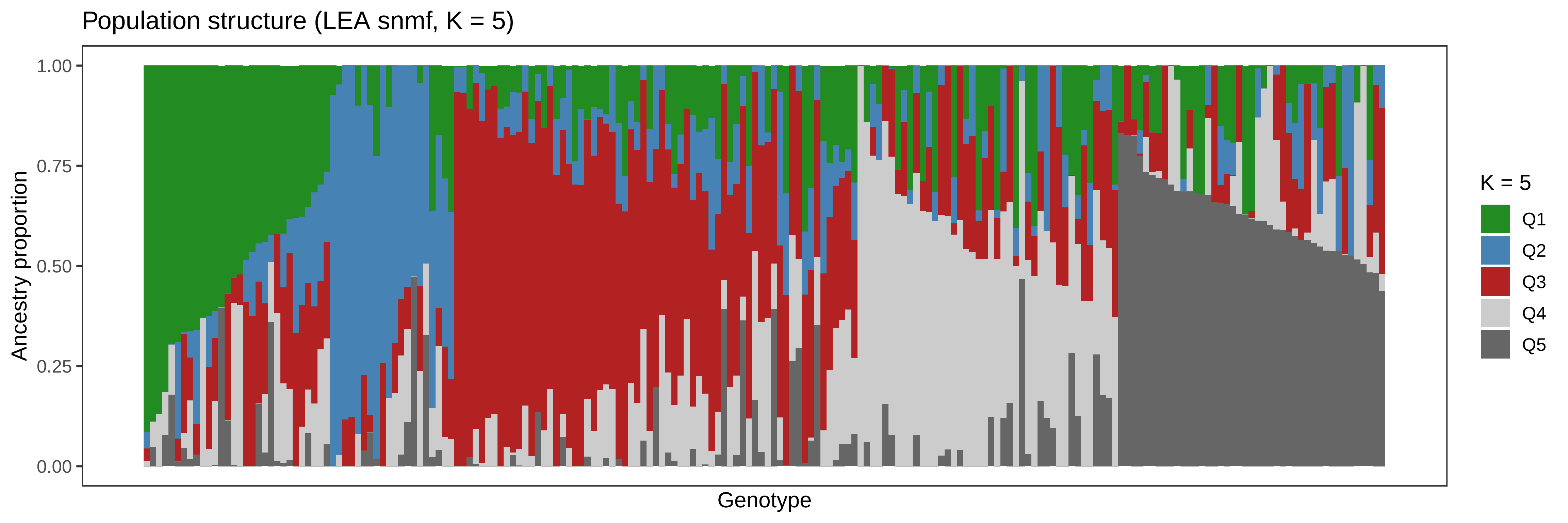
Population structure (LEA snmf, K = 5)
Ancestry proportion
1.00
0.75
0.50
0.25
0.00
Genotype
K = 5
Q1
Q2
Q3
Q4
Q5
