# Supplementary material for: Late blight resistance of Julius Kühn Institute pre-breeding potato clones: a genome-wide association study
Source: BMC Plant Biol. 2026 Jun 17;26:1046. doi: 10.1186/s12870-026-09266-3 (PMC13273995; doi:10.1186/s12870-026-09266-3)
Supplement: Supplementary file 4 — Additional file 4: Figures S3—S6: Residual diagnostics for the detached leaf assay, tuber slice test, rAUDPC, and ∆rAUDPC fitted separately for each genotype group (Non-carriers, Chr9, Chr11). For each combination, histograms of residuals (top), residuals versus fitted values (middle), and quantile–quantile plots (bottom) are shown. [file 12870_2026_9266_MOESM4_ESM.pdf]

Detached leaf assay

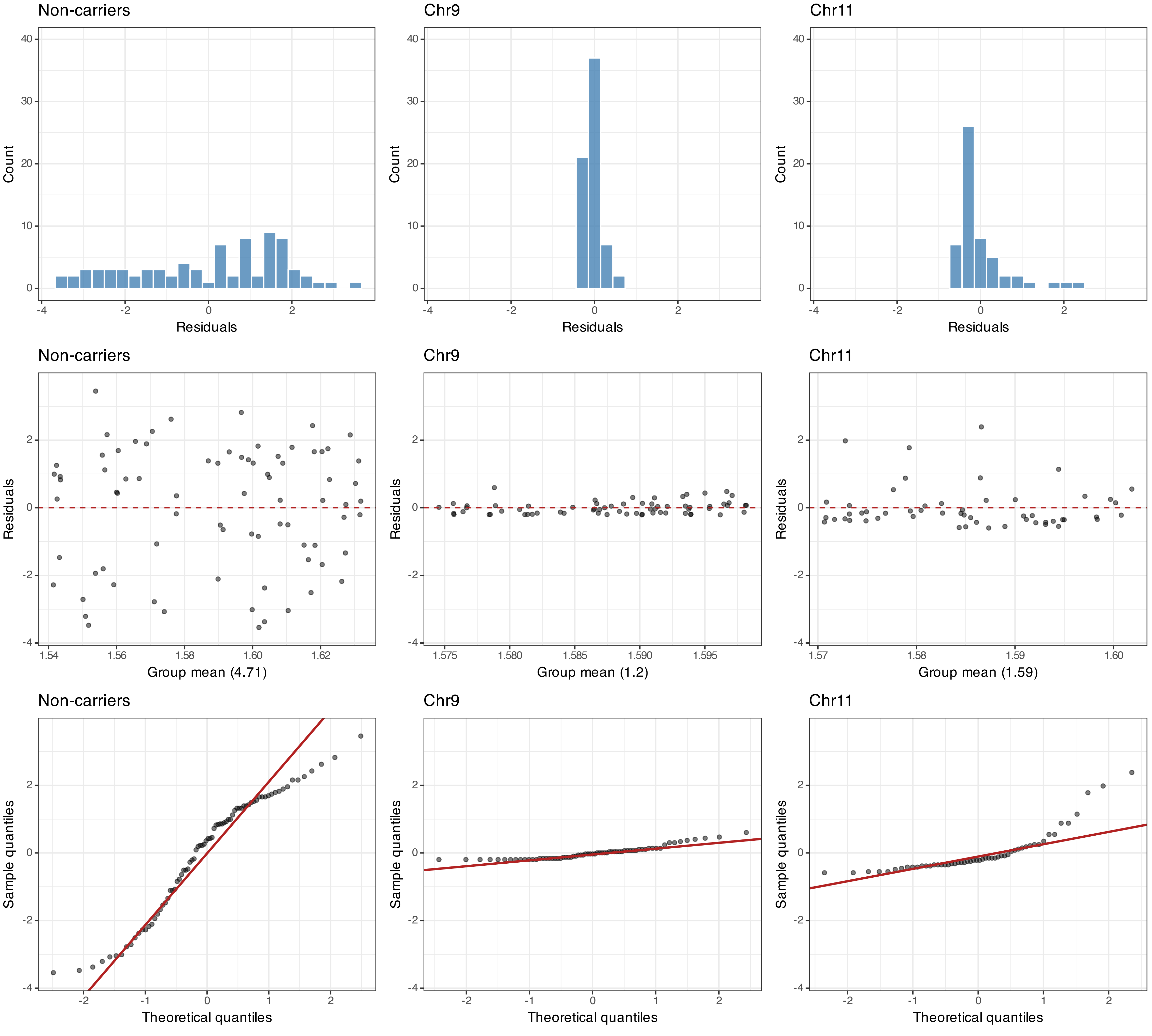

Tuber slice test

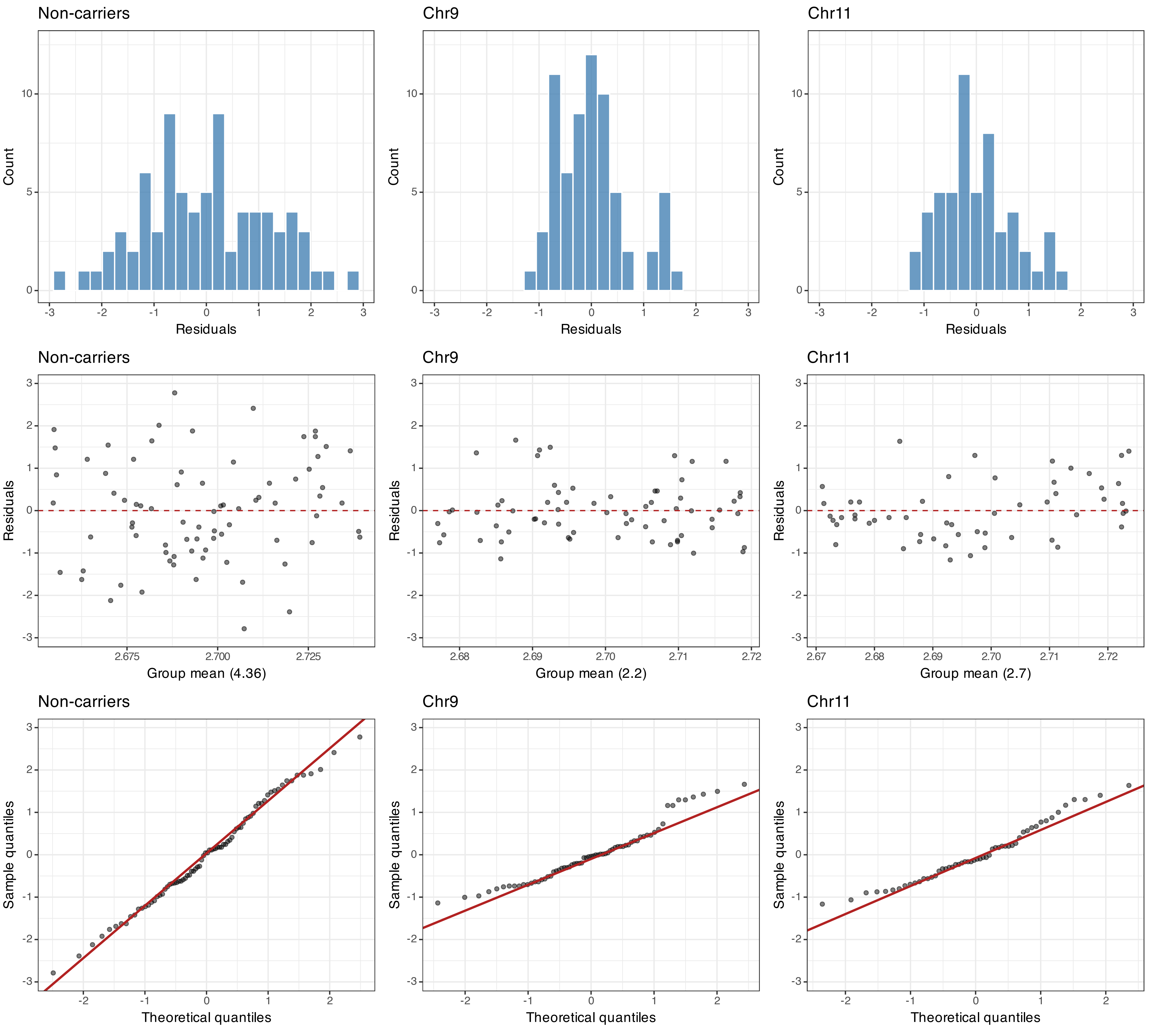

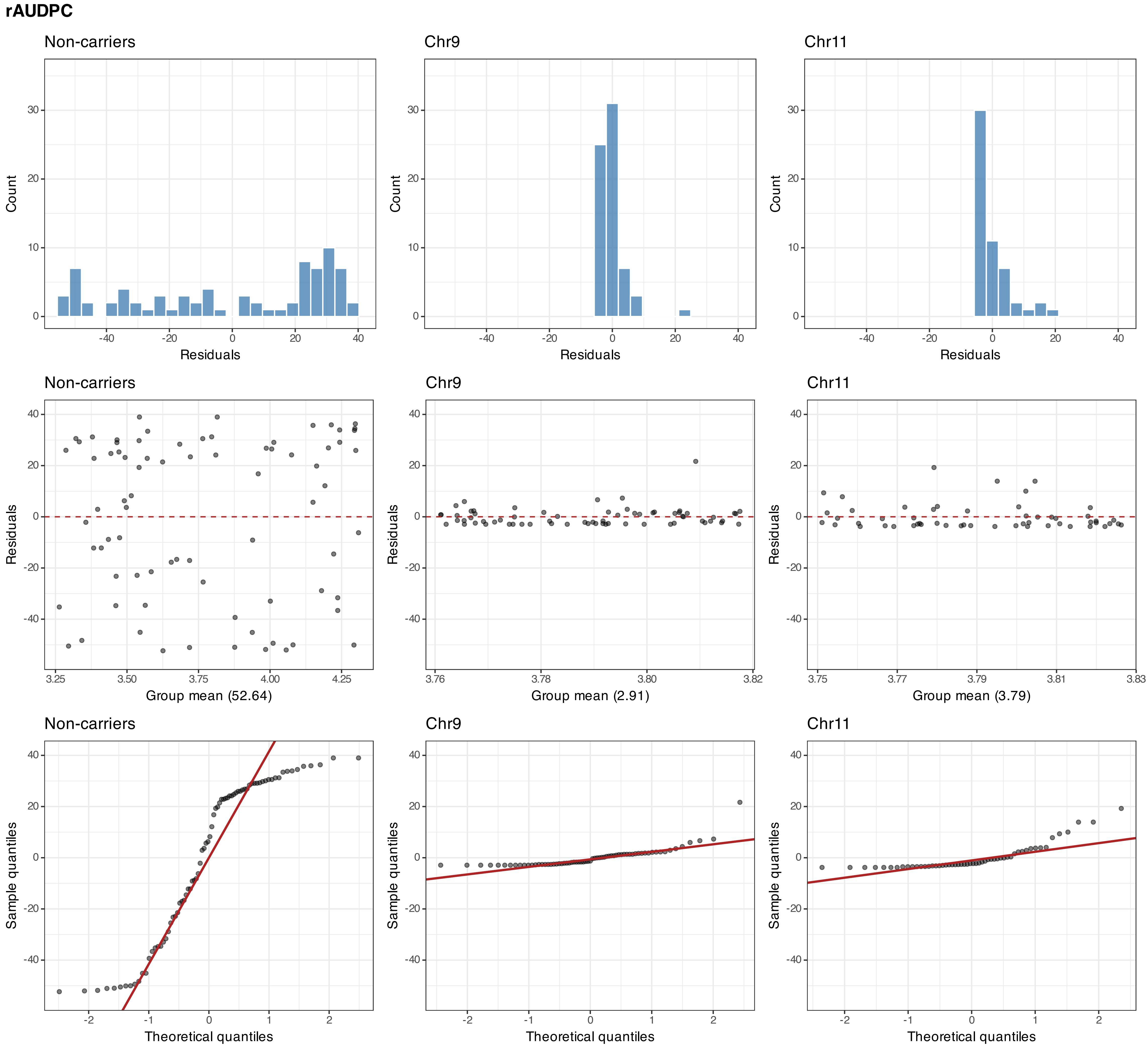
rAUDPC
Non-carriers
Chr9
Chr11
Count
Residuals
Group mean (52.64)
Group mean (2.91)
Group mean (3.79)
Sample quantiles
Theoretical quantiles

ΔrAUDPC

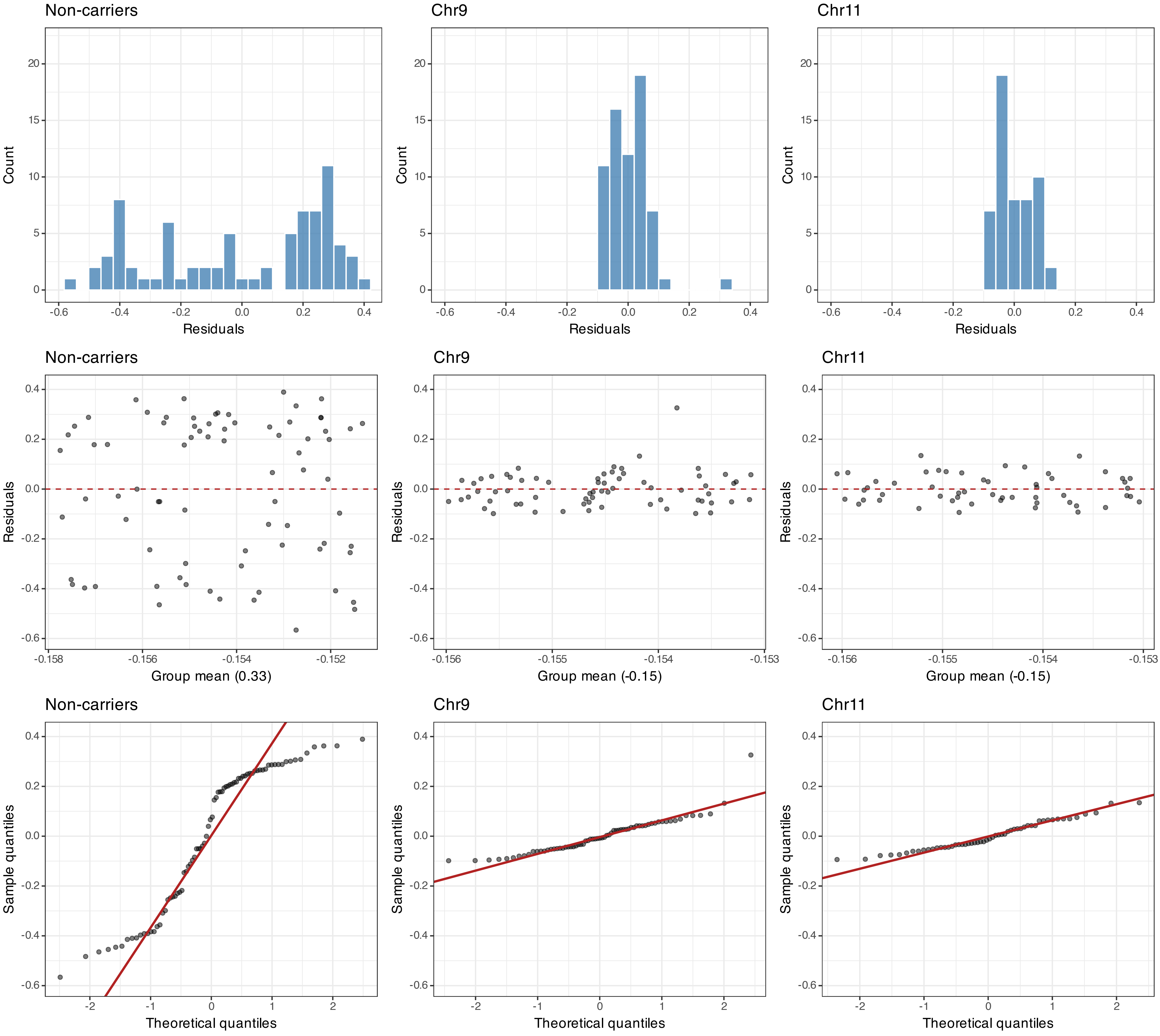
Non-carriers
Chr9
Chr11
Count
Residuals
Group mean (0.33)
Group mean (-0.15)
Sample quantiles
Theoretical quantiles
