# Supplementary figures and images for: Late blight resistance of Julius Kühn Institute pre-breeding potato clones: a genome-wide association study
Source: BMC Plant Biol. 2026 Jun 17;26:1046. doi: 10.1186/s12870-026-09266-3 (PMC13273995; doi:10.1186/s12870-026-09266-3)

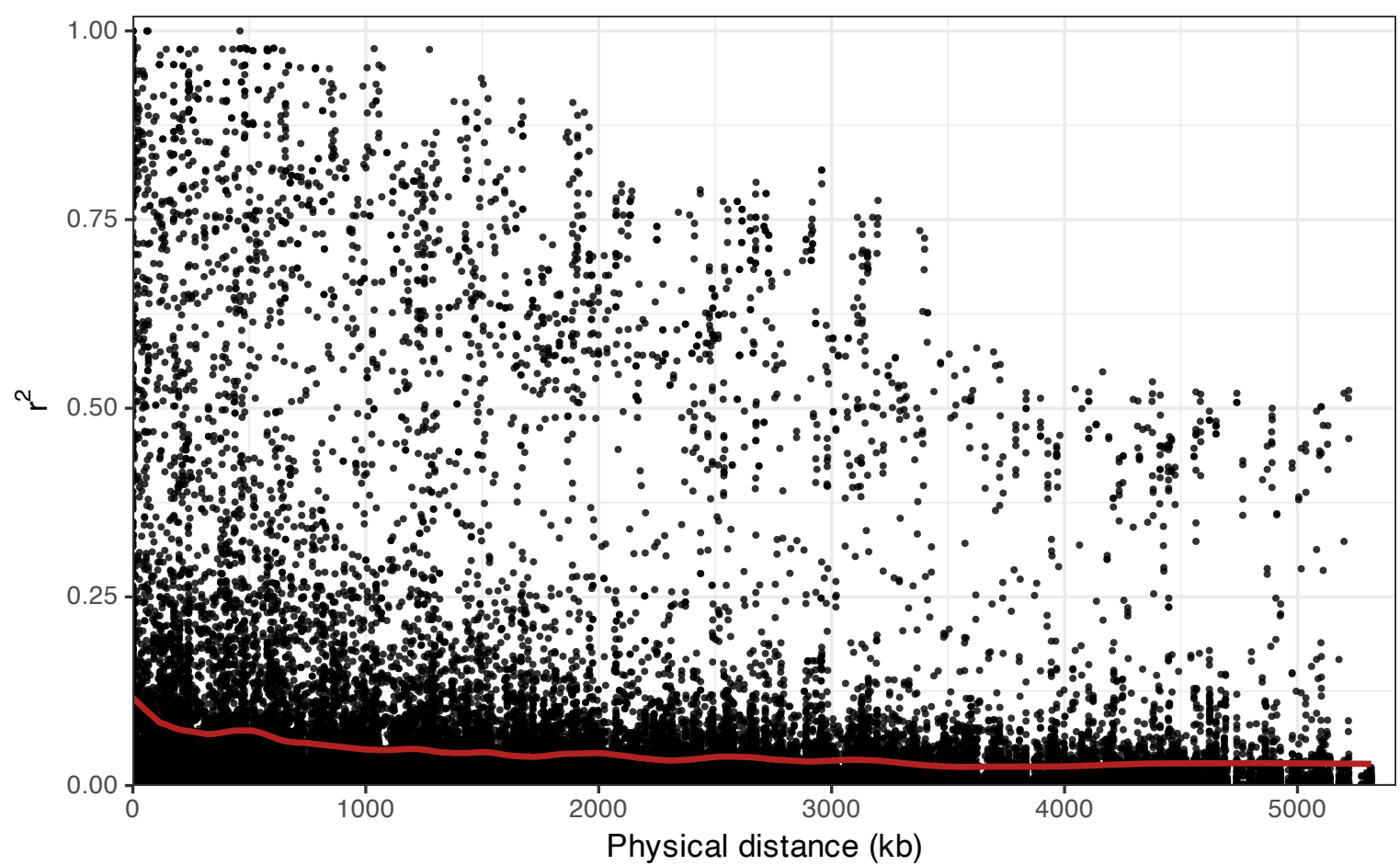

Supplement: Supplementary file 5 — Additional file 5: Figure S7: Linkage disequilibrium (r2) decay across the Chr11 resistance region (0.16–5.48 Mb) among 353 markers. The LOESS curve (red line) illustrates the decay trend. [file 12870_2026_9266_MOESM5_ESM.pdf]

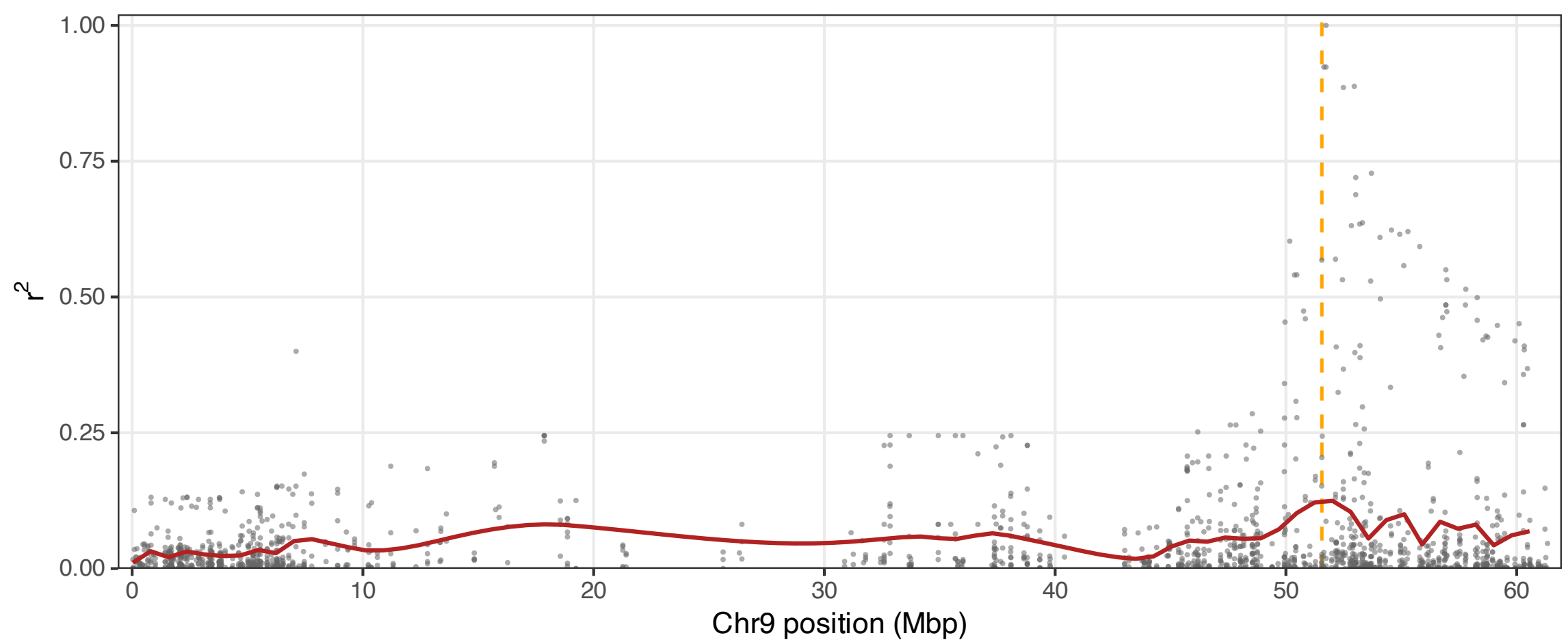

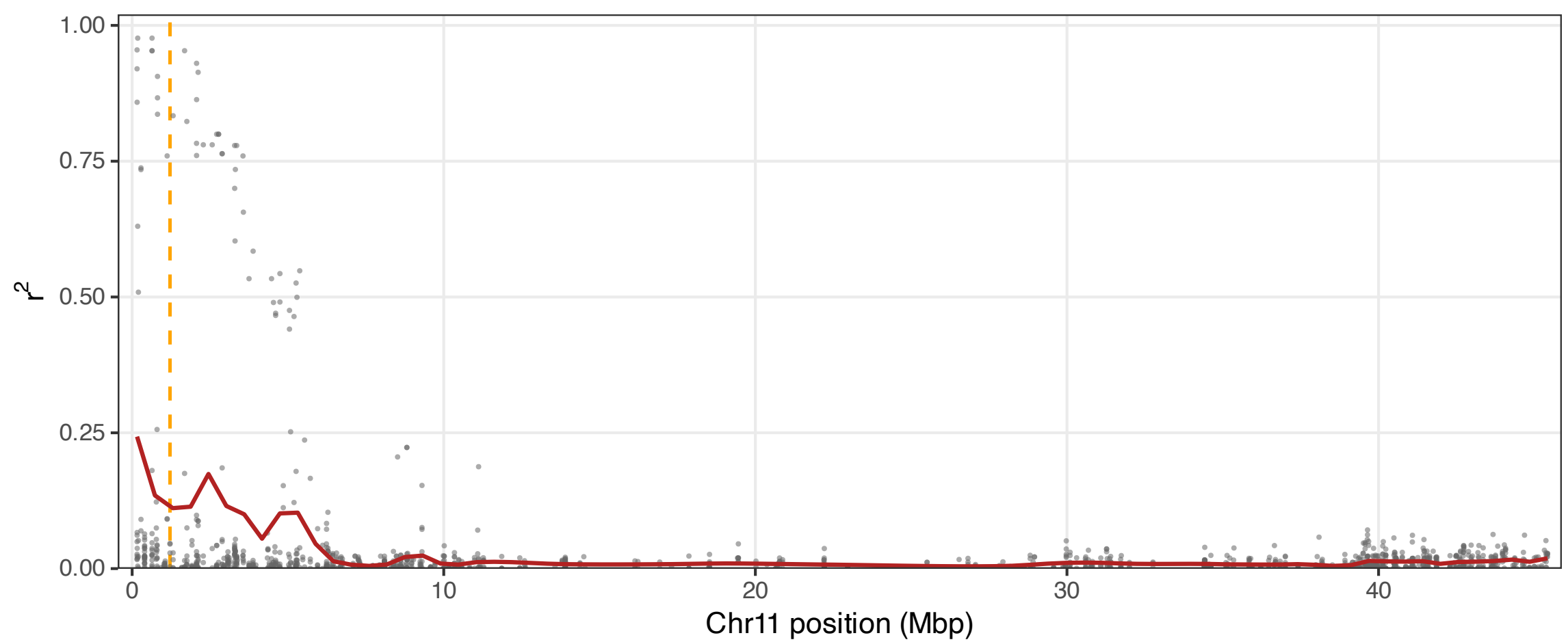

Supplement: Supplementary file 7 — Additional file 7: Figures S10, S11: LD profiles of the most significant markers on chromosomes 9 and 11 (indicated by dashed orange lines) against all chromosome 9 and 11 markers, respectively. Each point represents the r2 value between the anchor marker and another chromosome 9 or 11 marker. The red line shows a LOESS-smoothed trend. [file 12870_2026_9266_MOESM7_ESM.pdf]
