# Supplementary material for: Late blight resistance of Julius Kühn Institute pre-breeding potato clones: a genome-wide association study
Source: BMC Plant Biol. 2026 Jun 17;26:1046. doi: 10.1186/s12870-026-09266-3 (PMC13273995; doi:10.1186/s12870-026-09266-3)
Supplement: Supplementary file 6 — Additional file 6: Figures S8, S9: Manhattan plots after including 11_1214801 as a fixed covariate (first page) and after removing the marker effect from each phenotypic trait by linear regression (second page) for the traits detached leaf assay, tuber slice test, rAUDPC, and maturity-corrected relative area under disease progress curve (∆rAUDPC). [file 12870_2026_9266_MOESM6_ESM.pdf]

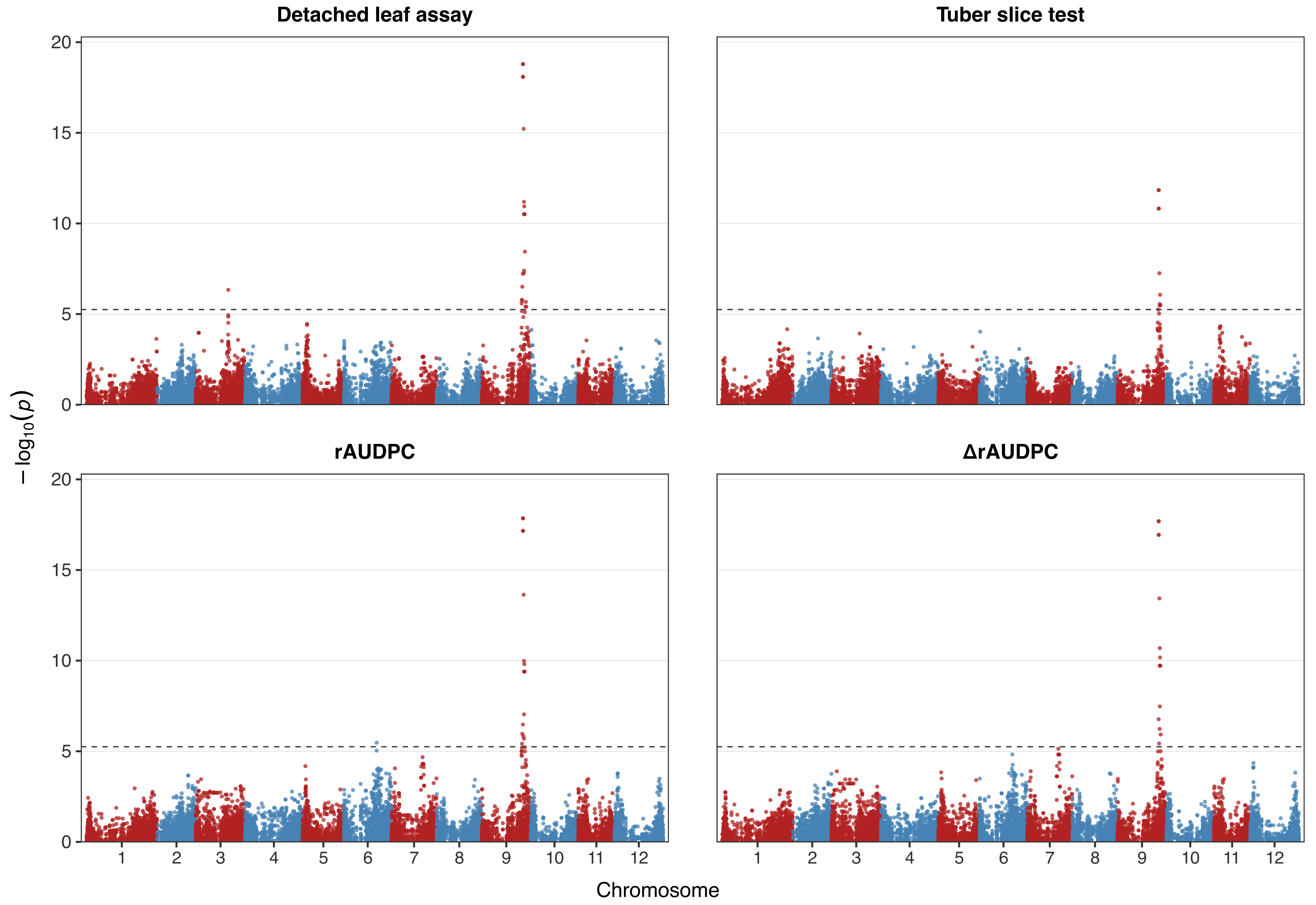

Detached leaf assay
Tuber slice test
rAUDPC
ΔrAUDPC
−log10(p)
Chromosome
0
5
10
15
20
1
2
3
4
5
6
7
8
9
10
11
12

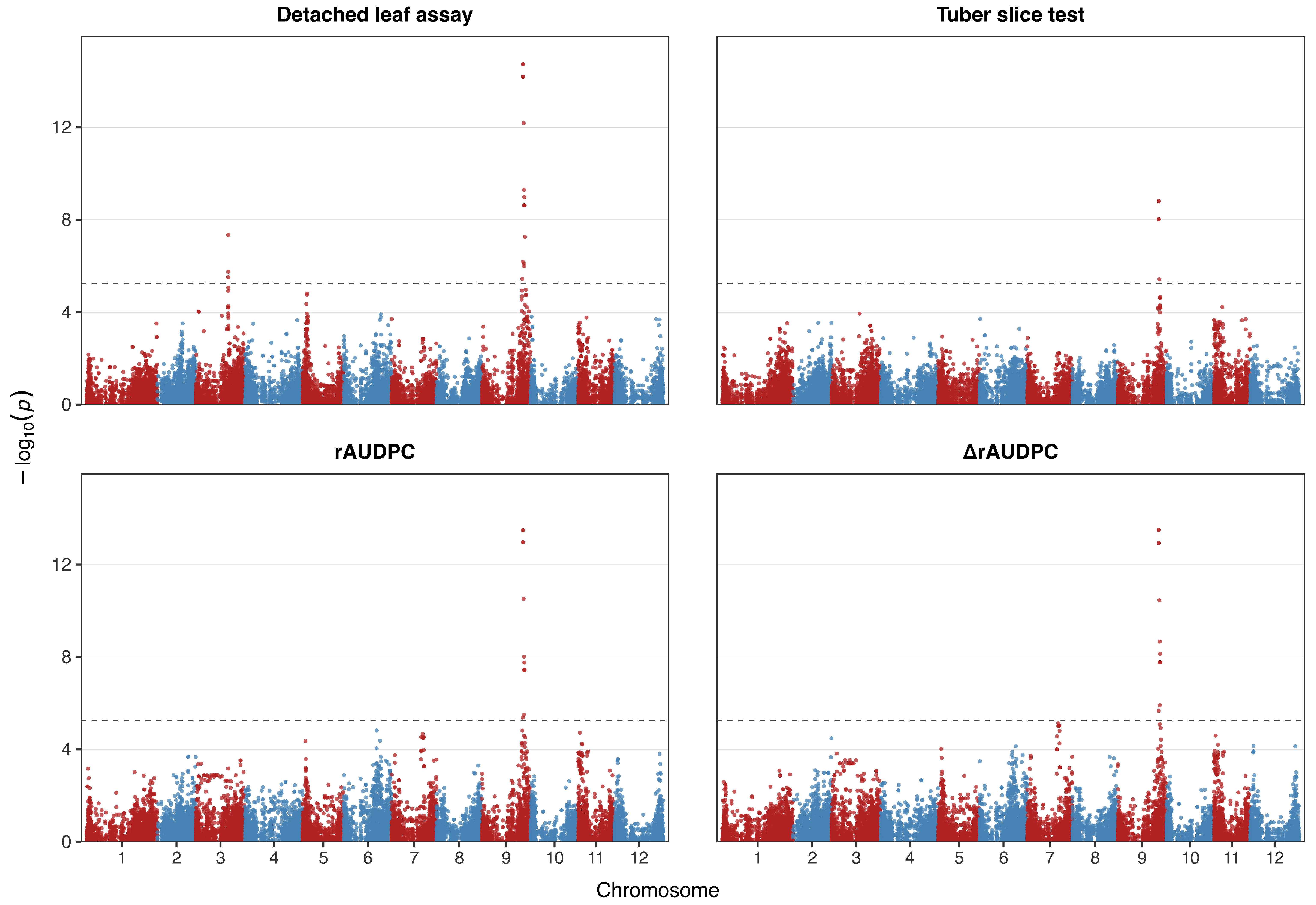
Detached leaf assay
Tuber slice test
rAUDPC
ΔrAUDPC
−log10(p)
Chromosome
0
4
8
12
1
2
3
4
5
6
7
8
9
10
11
12
